# Supplementary material for: Proteomic and Transcriptomic Patterns during Lipid Remodeling in Nannochloropsis gaditana
Source: Int J Mol Sci. 2020 Sep 22;21(18):6946. doi: 10.3390/ijms21186946 (PMC7554720; doi:10.3390/ijms21186946)
Supplement: Supplementary file 1 [file ijms-21-06946-s001.zip › MDPI.nannochloropsis.mdpi.two.supp.files.zipped.R2/MDPI.nannochloropsis.supplementary.data.pdf]

International Journal of
Molecular Sciences

MDPI

Supplementary data

Proteomic and transcriptomic patterns during lipid remodeling in *Nannochloropsis gaditana*

Chris J. Hulatt [1,*], Irina Smolina [1], Adam Dowle [2], Martina Kopp [1], Ghana K. Vasanth [1], Galice Hoarau [1], René H. Wijffels [3] and Viswanath Kiron [1]

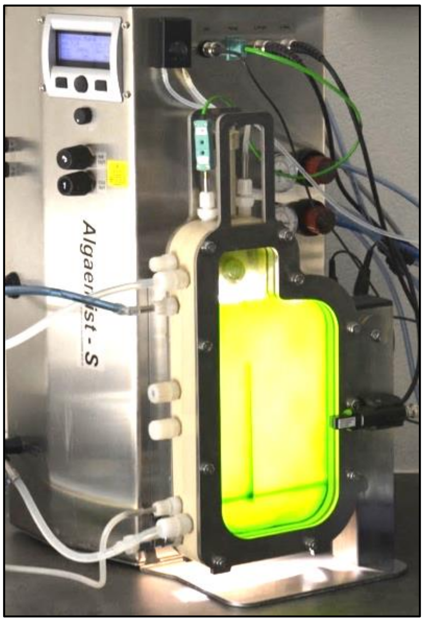

Proteomic analysis

Supplementary Table 1. The cultivation sequence of samples/treatments in the two photobioreactors was designed to prevent bias across individual photobioreactor units or time periods. "Batch" refers to the order in which cultivations were conducted. Two photobioreactors "photobioreactor ID" were run simultaneously and each "treatment" was assigned as indicated.

Batch	photobioreactor ID	treatment
1	A	Control
1	B	Nitrogen
2	A	Phosphorus
2	B	Control
3	A	Nitrogen
3	B	Phosphorus
4	A	Control
4	B	Nitrogen
5	A	Phosphorus
5	B	Control
6	A	Nitrogen
6	B	Phosphorus

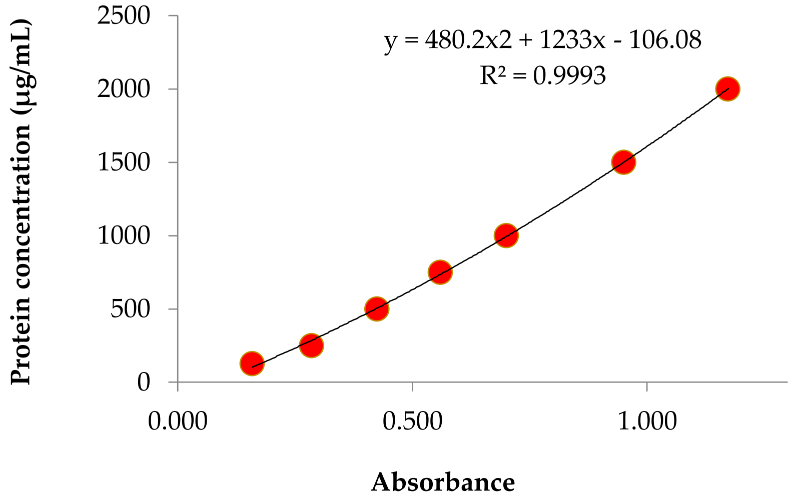

Supplementary Figure 1. Calibration plot from the BCA assay used for quantitation and normalization of protein extracted from each sample. A standardized 95.1 µg of protein from each sample was used for TMT LC-MS/MS analysis.

Supplementary Table 2. Peptides were labelled with TMT 10-plex reagents (Thermo Scientific) as detailed in the manufacturer's protocol. The labels were paired with the samples as below, where Sample represents the protein sample id (P1 to P10) and the label is the specific index provided by the manufacturer.

Sample	Treatment	Label
P1	Control	127N
P2	Nitrogen	128N
P3	Control	127C
P4	Nitrogen	128C
P5	Control	129N
P6	Nitrogen	130N
P7	Control	129C
P8	Nitrogen	130C
P9	Phosphorus	126
P10	Phosphorus	131

1.2 Transcriptome sequencing

A number of genome assemblies are available for different strains of *Nannochloropsis*. In order to obtain the best results from our transcriptomic data, we first confirmed the strain we used in the experiments using the mitogenome and 18S ribosomal genes and then identified two genome sequences that could potentially be used for gene expression analysis. We compared the results of transcript alignment between the two genome assemblies to determine the best option for our data.

1.2.1 Genetic confirmation of the experimental strain.

We confirmed the identity of the experimental strain using two approaches. First, reads from several libraries were aligned to mitochondrial genome sequences of *N. gaditana* B31 and CCMP526 strains using Bowtie2. The alignment to each mitochondrial genome was visualized using Integrative Genomics Viewer. Across more than 30 kb of mitochondrial genome, alignments to the CCMP526 genome showed no SNPs, while alignments to B31 genome showed 20 SNPs with alternative alleles being identical to the CCMP526 strain. The second approach included alignment of nuclear 18S ribosomal RNA from the experimental strain and from *N. gaditana* reference genomes B31 and CCMP526, as well as 4 other *Nannochloropsis* species: *N. salina* (accession numbers are AF045048.1 and AF045050.1), *N. granulata* (U41092.1, AF045041.1 and AF045042.1), *N. oceanica* (JF489982.1), and *N. oculata* (AF045044.1 and AF045045.1). Sequences were aligned using the ClustalW algorithm with default parameters in Geneious (Bio Matters). Multiple sequence alignment was used to build a Neighbour-Joining tree using Tamura-Nei distance model as implemented in Geneious. Both approaches confirmed that the strain used in this study is *N. gaditana* CCMP526, and that both *N. gaditana* B31 and CCMP526 are very similar amongst strains of *Nannochloropsis* (Supplementary Figure 2).

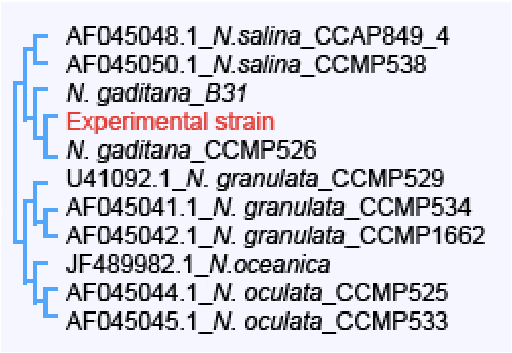

Supplementary Figure 2. Multiple sequence alignment of 18S sequences amongst ten strains of *Nannochloropsis*, including the experimental strain (red) that was cultivated in our photobioreactors.

1.2.2 Transcript read mapping

Due to the similarity between the CCMP526 and B31 strains, we decided to map reads to both genome assemblies and compare the results. We found that the more recent B31 genome (2014) provided measurably higher transcript mapping statistics (Supplementary Table 3) than the original CCMP526 assembly (2011). Taking into account the genetic similarity, the read mapping statistics and the protein databases, we chose to use the B31 mapping results in the manuscript.

Supplementary Table 3. Comparison of our sequenced libraries mapped to the genomes of strains CCMP526 (assembly ASM24072v1) and B31 (assembly NagaB31_1.0). The library index corresponds to the treatment (C= control, N= nitrogen, P= phosphorus), the time (3 or 5 days) and the replicate culture (A,B,C,D), e.g. the first entry "C_3_A" is a control sample from day 3, replicate A.

Library index	Raw reads	**B31 assembly**		**CCMP526 assembly**	
		Uniquely mapped reads	% uniquely mapped reads	Uniquely mapped reads	% uniquely mapped reads
C_3_A	22370337	18872400	84.8	15794981	71.0
C_3_B	18628638	16298460	88.0	13504147	72.9
C_3_C	19498430	17011555	87.8	14034465	72.4
C_3_D	4387222	3850742	88.2	3193371	73.1
C_5_A	17695722	14462685	82.3	12108169	68.9
C_5_B	18716317	16469217	88.5	13642309	73.3
C_5_C	19314569	16945821	88.2	14007109	72.9
C_5_D	15871684	13705824	86.8	11504378	72.9
N_3_A	19490270	16425553	84.8	13614231	70.3
N_3_B	19142078	16917222	88.9	14002819	73.6
N_3_C	18703611	16372489	88.0	13437923	72.2
N_3_D	17877643	15841284	89.1	12993388	73.1
N_5_A	18765398	14850840	79.7	12024143	64.5
N_5_B	17494433	15487549	89.0	12846836	73.8
N_5_C	26281440	22834429	87.4	18711245	71.6
N_5_D	18269440	16150479	88.9	13148045	72.3
P_3_A	18513837	16014709	86.9	13627442	74.0
P_3_B	18850927	16489667	87.9	13746148	73.3
P_3_C	18746741	16368332	87.8	13623486	73.1
P_3_D	19954394	17423274	87.8	14512272	73.1
P_5_A	17934875	15415194	86.4	12959870	72.6
P_5_B	18120731	15603526	86.6	13211108	73.3
P_5_C	20407306	17533555	86.4	14565102	71.8
P_5_D	17745886	15207838	86.2	12631985	71.6
Average	18449247	15939693	86.9	13226873	72.2

1.3 Principal component analysis (PCA)

Principal component analysis was used to initially visualize the clustering amongst experimental treatments in the protein and transcript datasets. Supplementary Figure 3a represents the clustering amongst the 10 protein samples from day 3, indicating distinct clustering of N- samples but weaker demarcation between C and P- treatments. Supplementary Figure 3b shows a similar pattern for the transcript data at day 3, whilst at day 5 (Supp. Fig. 3c) the pattern in the transcript data strengthens and each of the three treatments cluster separately.

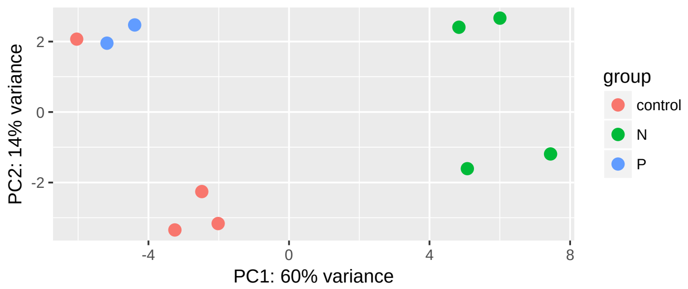

(a)

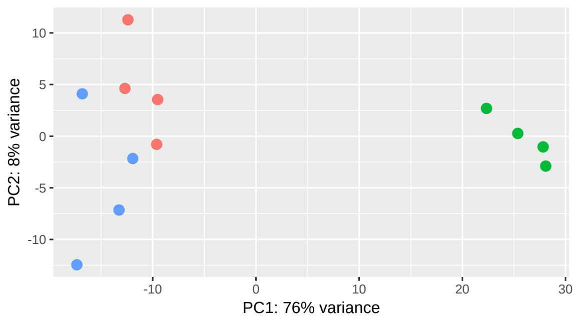

(b)

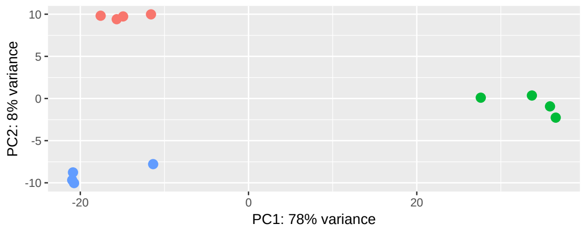

(c)

Supplementary Figure 3. Clustering of experimental samples from control, nitrogen starved and phosphorus starved treatments represented by principal component analysis (PCA). (a) Protein samples from day 3 (n = 10). (b) Transcript samples from day 3 (n = 12) and (c) Transcript samples from day 5 (n = 12).

1.4 Protein-transcript correlations

In the manuscript we present three different approaches for characterizing the relationship between transcript and protein expression from the same biological samples. The following provides extra details for the analysis shown in manuscript Figure 3.

1.4.1 Association of proteins and transcripts

We associated proteins and their transcripts together using their unique accession numbers. Although in total 3,423 proteins were identified in the proteomics analysis, some of these were CCMP526 accessions and so did not have matching transcripts. The data in the correlation analysis are therefore all based on the protein and transcript accessions from the B31 genome assembly.

1.4.2 Linear mixed-effects model of RPKM vs Mol% (Manuscript Figure 3b)

Our TMT experiment was designed to identify differential expression across treatments by comparing the abundance of different reporter ions in a multiplexed sample. Whilst the TMT reporter ion intensity roughly scales with (and correlates with) protein abundance, it does not provide absolute quantitation of proteins. This is due to the nature of the labelling of peptides because, although the reporter ion intensity provides very accurate quantitation for the same peptide in different samples, the reporter ion intensity is only semi-quantitative across different types of peptides. To improve our analysis in Figure 3b, we therefore used the protein abundance in multiplexed samples (PAMUS) approach described in the manuscript. For transcript data, we used the mapped read counts per kilobase of exon, per million reads (RPKM).

To fit the regression line, a linear mixed-effects model was used to account for the data structure, where matching protein and transcript data from ten experimental cultures were available. The data were modelled using the R package *nlme*, fitting a random slope and random intercept for each of the ten samples. The model, following *nlme* notation, is shown in eq. 1, after Pinheiro & Bates (2006).

lme(logRPKM ~ logMol%, random= ~1 + logMol% | replicate) (1)

Where "logRPKM" is the natural logarithm of transcript counts in units RPKM and "logMol%" is the natural logarithm of protein abundance in Mol%. The "replicate" term is the individual turbidostat cultivation ($n = 10$). The slopes and intercepts of the linear relation between log(RPKM) and log(Mol%) for each replicate cultivation are shown in Table S4. In addition, the R_2 correlation coefficients for each are presented, where the mean $R_2 = 0.31$.

Supplementary Table 4. The slopes and intercepts of the linear relation between transcript log(RPKM) and protein log(Mol%) for each sample. The R^2 and number of data points included is also presented.

Replicate cultivation	Intercept	Slope	R^2	n
C1	-7.78	0.645	0.34	2,540
C2	-7.49	0.607	0.32	2,546
C3	-7.90	0.661	0.34	2,540
C4	-7.53	0.611	0.30	2,541
N1	-7.71	0.624	0.27	2,526
N2	-7.78	0.660	0.30	2,526
N3	-8.10	0.691	0.30	2,513
N4	-7.81	0.662	0.30	2,518
P1	-7.69	0.621	0.29	2,568
P2	-7.84	0.656	0.35	2,530

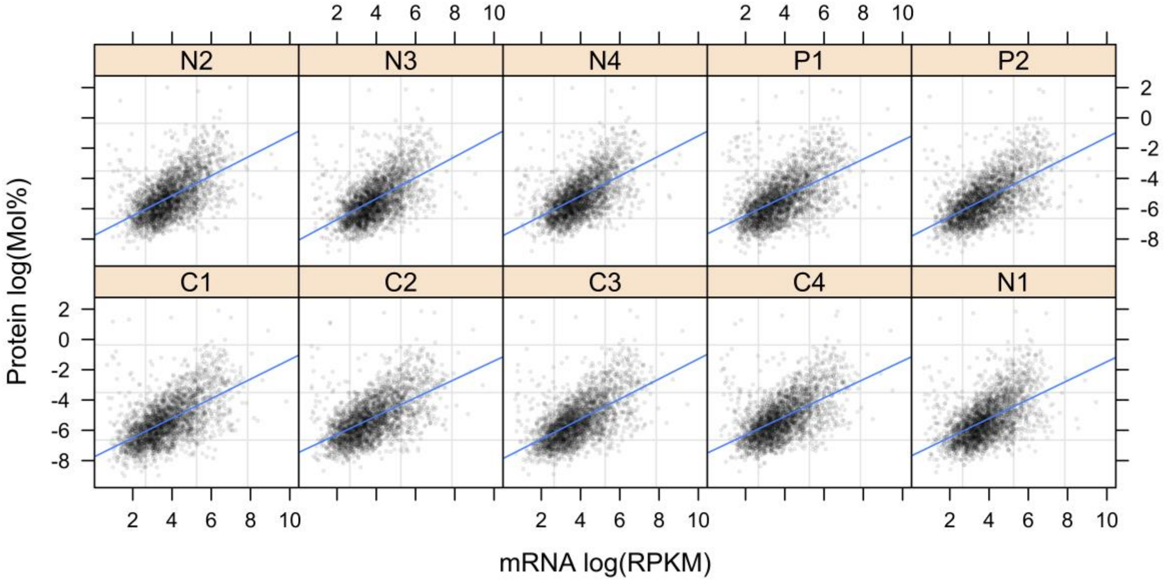

Supplementary Figure 4. Panel plot showing the correlation between protein abundance (logMol%) and transcript abundance (logRPKM) for each of the ten individual cultivations.

1.4.3 Linear models fitted to each protein/transcript accession (Manuscript Figures 3c and 3d)

Our third method captures the correlation between protein abundance and transcript abundance for each accession separately. The data describes the heterogeneity of correlations across the proteome. We again use log(RPKM) transcript counts as the measure of mRNA abundance, but since these are correlations within individual proteins/genes, we did not apply any emPAI adjustments. Instead, we simply used the normalized TMT reporter ion intensities and acknowledge that the absolute values of the slopes will vary between protein/transcript accessions, depending on individual peptide/protein response factors. In total, 2,576 simple linear models were fitted and R^2 values were calculated. A small subset of these linear models are shown as examples in Supplementary Figure 5.

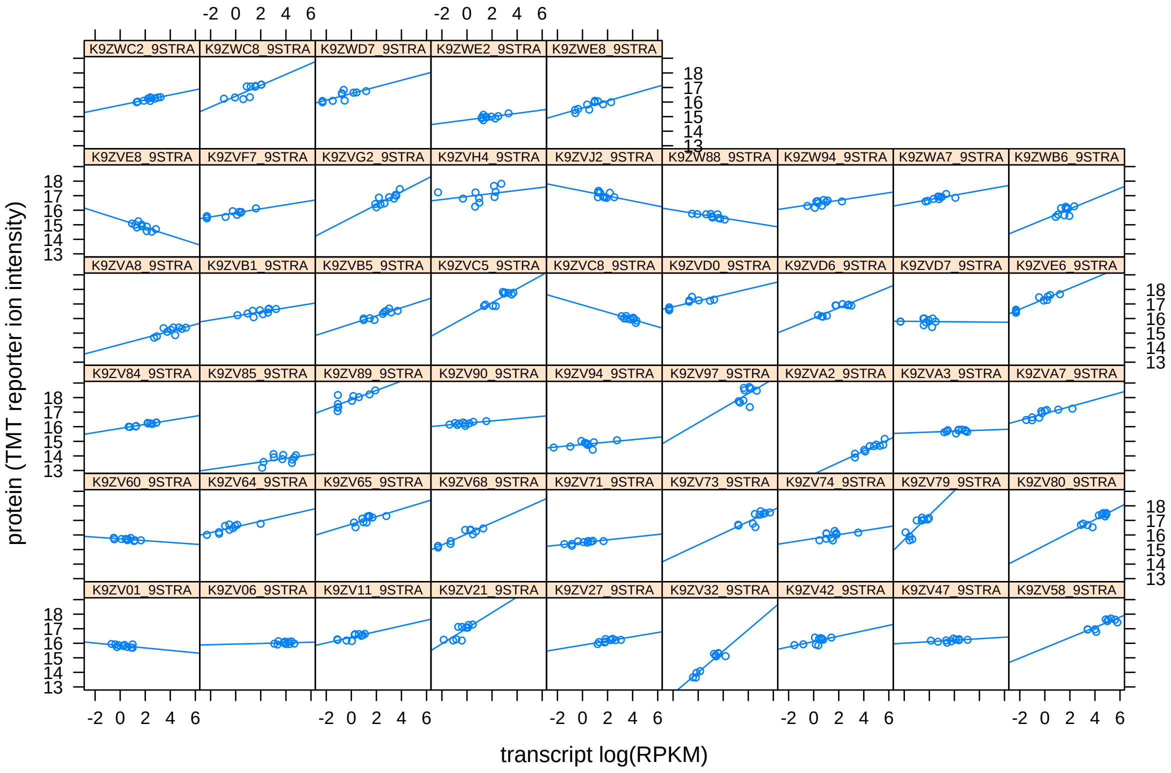

Supplementary Figure 5. A small subset of 50 linear regression models (out of 2,576) provided as an example of the method. Each panel represents one gene/protein. Since there were 10 TMT labels, there are a maximum of n=10 data points in each correlation. On the x-axis is the log(RPKM) transcript counts, whilst the y-axis is the normalized TMT reporter ion intensity directly from the LC-MS/MS analysis. The data is summarized in the manuscript by the R^2 values and the slope (Figure 3c, 3d).

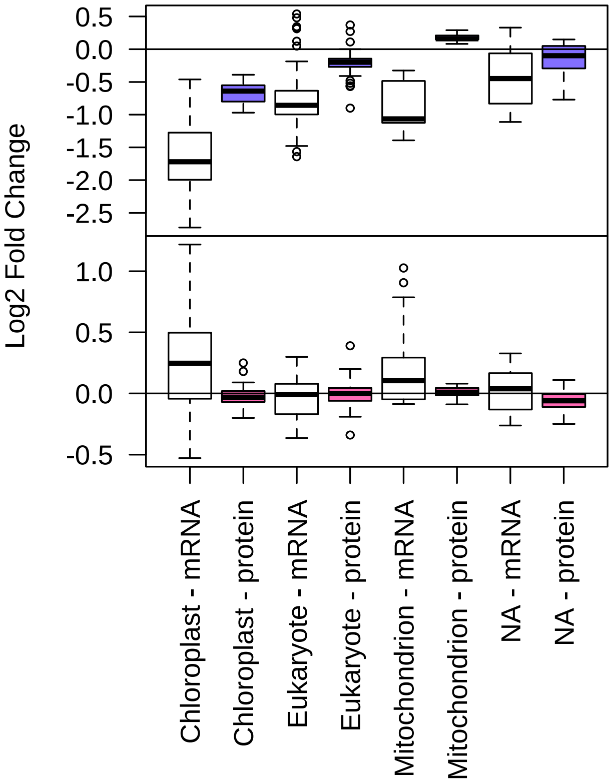

Supplementary Figure 6. Fold changes of ribosomal proteins and ribosomal mRNA transcripts in N- (upper panel) and P- (lower panel) treatments relative to controls, after 3 days of treatment. Ribosomal proteins and transcripts are grouped as follows; plastid (prokaryotic, including 30S & 50S), mitochondrion, eukaryote (including cytoplasm and endoplasmic reticulum, 40S & 60S) and NA (not annotated).

Supplementary Table 5. Differential expression of proteins annotated as amine oxidases in N-/Control and P-/Control treatments. Proteins determined significantly differently expressed at the Benjamini-Hochberg adjusted p-value thresholds are indicated with (**), those significant at the 5% level are indicated (*).

Condition	Name	UniProt Accession Number	Molecular Weight	No. quantified spectra	L_2fc	p-value
N/Control	Amine oxidase	W7U0L2_9STRA	87 kDa	12	0.03	0.28
N/Control	Amine oxidase	W7U1W2_9STRA	64 kDa	15	0.16	0.001**
N/Control	Amine oxidse	W7TFN3_9STRA	75 kDa	18	1.38	0.0001**
N/Control	Amine oxidase	W7TES8_9STRA	63 kDa	6	0.21	0.074
N/Control	Amine oxidase	K8YW47_NANGC	90 kDa	10	0.11	0.18
N/Control	Amine oxidase	I2CR86_NANGC	20 kDa	6	0.27	0.00027**
N/Control	Amine oxidase (fragment)	I2CPM9_NANGC	31 kDa	7	0.16	0.13
P/Control	Amine oxidase	W7U0L2_9STRA	87 kDa	12	0.07	0.11
P/Control	Amine oxidase	W7U1W2_9STRA	64 kDa	15	0.05	0.55
P/Control	Amine oxidase	W7TFN3_9STRA	75 kDa	18	-0.32	0.0001**
P/Control	Amine oxidase	W7TES8_9STRA	63 kDa	6	-0.27	0.019*
P/Control	Amine oxidase	K8YW47_NANGC	90 kDa	10	0.01	0.72
P/Control	Amine oxidase	I2CR86_NANGC	20 kDa	6	-0.01	0.89
P/Control	Amine oxidase (fragment)	I2CPM9_NANGC	31 kDa	7	0.09	0.16

Supplementary Table 6. Differential expression of proteins annotated as desaturases in N-/Control and P-/Control treatments. Proteins determined significantly differently expressed at the Benjamini-Hochberg adjusted p-value thresholds are indicated with (**), those significant at the 5% level are indicated (*).

Condition	Name	UniProt Accession Number	Molecular Weight	No. quantified spectra	L_2fc	p-value
N/Control	Delta 5 fatty acid desaturase	K8YSX2_NANGC	54 kDa	5	-0.95	0.0001**
N/Control	Fatty acid desaturase type 2	W7UCJ8_9STRA	38 kDa	32	0.09	0.016**
N/Control	Glycerolipid omega-3 fatty acid desaturase (Fragment)	I2CR09_NANGC	51 kDa	3	-0.53	0.001**
N/Control	Omega-6 fatty acid desaturase delta-12	K8YR13_NANGC	51 kDa	3	-0.37	0.005**
N/Control	Phytoene desaturase	W7TCB4_9STRA	66 kDa	39	-0.16	0.001**
N/Control	Stearoyl-desaturase 5	W7TAD9_9STRA	39 kDa	7	-0.19	0.006**
N/Control	Zeta-carotene desaturase	W7UB54_9STRA	67 kDa	32	-0.33	0.0001**
P/Control	Delta 5 fatty acid desaturase	K8YSX2_NANGC	54 kDa	5	0.03	0.75
P/Control	Fatty acid desaturase type 2	W7UCJ8_9STRA	38 kDa	32	-0.01	0.59
P/Control	Glycerolipid omega-3 fatty acid desaturase (Fragment)	I2CR09_NANGC	51 kDa	3	0.18	0.13
P/Control	Omega-6 fatty acid desaturase delta-12	K8YR13_NANGC	51 kDa	3	-0.04	0.82
P/Control	Phytoene desaturase	W7TCB4_9STRA	66 kDa	39	-0.01	0.34
P/Control	Stearoyl-desaturase 5	W7TAD9_9STRA	39 kDa	7	0.05	0.74
P/Control	Zeta-carotene desaturase	W7UB54_9STRA	67 kDa	32	-0.04	0.13

Supplementary Table 7. Fold changes and adjusted p-values of diacylglycerol acyltransferase transcripts, as determined by *DESeq2* analysis of RNA-seq data. The corresponding Protein ID is shown for cross-referencing to the proteome data.

	gene ID	Protein ID	Gene name	L_2fc	p-adj
N/Control	Naga_100006g86	W7U9S5_9STRA	Diacylglycerol acyltransferase family protein	1.000	1.32E-22
N/Control	Naga_100010g4	W7U2S6_9STRA	Diacylglycerol acyltransferase family protein	0.104	0.671
N/Control	Naga_100028g44	W7TT81_9STRA	Diacylglycerol acyltransferase family protein	-0.400	0.002
N/Control	Naga_100030g42	W7TTN1_9STRA	Diacylglycerol o-acyltransferase 2	-1.145	8.41E-16
N/Control	Naga_100071g18	W7TPK1_9STRA	Diacylglycerol o-acyltransferase 2	0.394	0.002
N/Control	Naga_100343g3	W7T9Y9_9STRA	Diacylglycerol acyltransferase type 2	0.965	1.12E-09
N/Control	Naga_100682g2	W7T2V9_9STRA	Diacylglycerol acyltransferase	-0.163	0.337
N/Control	Naga_101968g1	W7TT63_9STRA	Diacylglycerol acyltransferase	-0.149	0.405
P/Control	Naga_100006g86	W7U9S5_9STRA	Diacylglycerol acyltransferase family protein	-0.122	0.645
P/Control	Naga_100010g4	W7U2S6_9STRA	Diacylglycerol acyltransferase family protein	-0.062	0.922
P/Control	Naga_100028g44	W7TT81_9STRA	Diacylglycerol acyltransferase family protein	-0.121	0.712
P/Control	Naga_100030g42	W7TTN1_9STRA	Diacylglycerol o-acyltransferase 2	0.104	0.789
P/Control	Naga_100071g18	W7TPK1_9STRA	Diacylglycerol o-acyltransferase 2	-0.006	0.989
P/Control	Naga_100343g3	W7T9Y9_9STRA	Diacylglycerol acyltransferase type 2	0.211	0.574
P/Control	Naga_100682g2	W7T2V9_9STRA	Diacylglycerol acyltransferase	0.150	0.697
P/Control	Naga_101968g1	W7TT63_9STRA	Diacylglycerol acyltransferase	-0.294	0.406

Supplementary Table 8. Differential expression of proteins annotated as lipases in N-/Control and P-/Control treatments. Proteins determined significantly differently expressed at the Benjamini-Hochberg adjusted p-value thresholds are indicated with (**), those significant at the 5% level are indicated (*).

Condition	Name	UniProt Accession Number	Molecular Weight	No. quantified spectra	L_2fc	p-value
N/Control	Calcium-independent phospholipase a2-gamma	K8Z805_NANGC	111 kDa	2	0.02	0.42
N/Control	Cluster of Putative lipase (Fragment)	W7TJ50_9STRA	49 kDa	1	-0.11	0.56
N/Control	Esterase lipase thioesterase family protein	K8Z5U7_NANGC	45 kDa	7	-0.08	0.18
N/Control	Gdsl lipase acylhydrolase family protein	K8YWS8_NANGC	38 kDa	4	0.4	0.0001**
N/Control	Lipase class 3	W7U7Q2_9STRA	46 kDa	4	0.06	0.63
N/Control	Lipase domain-containing protein	W7TDJ3_9STRA	68 kDa	2	-0.21	0.17
N/Control	Lipase family protein	W7TUB0_9STRA	54 kDa	8	1.06	0.0001**
N/Control	Lipase	W7U0K9_9STRA	64 kDa	6	0.58	0.0001**
N/Control	Lipase, class 3 (Fragment)	W7TQA1_9STRA	53 kDa	8	0.02	0.46
N/Control	Lipase, class 3	W7U4G1_9STRA	68 kDa	6	0.18	0.001**
N/Control	Lipase, class 3	W7TNA7_9STRA	78 kDa	5	0.15	0.12
N/Control	Lysophospholipase II	K8Z7I5_NANGC	25 kDa	10	0.11	0.01**
N/Control	Lysophospholipase	W7U1W7_9STRA	112 kDa	5	0.11	0.14
N/Control	Lysophospholipase-like protein	W7TTI2_9STRA	47 kDa	8	0.29	0.00012**
N/Control	Monoglyceride lipase	W7TXG7_9STRA	40 kDa	4	-0.17	0.1
N/Control	Phospholipase-like protein	W7T9K1_9STRA	67 kDa	2	-0.11	0.12
N/Control	Triglyceride lipase	W7TSJ6_9STRA	86 kDa	4	0.21	0.038*
P/Control	Calcium-independent phospholipase a2-gamma	K8Z805_NANGC	111 kDa	2	-0.11	0.43
P/Control	Cluster of Putative lipase (Fragment)	W7TJ50_9STRA	49 kDa	1	-0.01	0.93
P/Control	Esterase lipase thioesterase family protein	K8Z5U7_NANGC	45 kDa	7	0.04	0.41
P/Control	Gdsl lipase acylhydrolase family protein	K8YWS8_NANGC	38 kDa	4	0.09	0.34
P/Control	Lipase class 3	W7U7Q2_9STRA	46 kDa	4	0.11	0.38
P/Control	Lipase domain-containing protein	W7TDJ3_9STRA	68 kDa	2	-0.17	0.14
P/Control	Lipase family protein	W7TUB0_9STRA	54 kDa	8	-0.32	0.001**
P/Control	Lipase	W7U0K9_9STRA	64 kDa	6	-0.11	0.34
P/Control	Lipase, class 3 (Fragment)	W7TQA1_9STRA	53 kDa	8	-0.03	0.79
P/Control	Lipase, class 3	W7U4G1_9STRA	68 kDa	6	-0.09	0.17
P/Control	Lipase, class 3	W7TNA7_9STRA	78 kDa	5	0.0	0.8
P/Control	Lysophospholipase II	K8Z7I5_NANGC	25 kDa	10	-0.03	0.5
P/Control	Lysophospholipase	W7U1W7_9STRA	112 kDa	5	-0.36	0.096
P/Control	Lysophospholipase-like protein	W7TTI2_9STRA	47 kDa	8	-0.04	0.54
P/Control	Monoglyceride lipase	W7TXG7_9STRA	40 kDa	4	-0.24	0.035*
P/Control	Phospholipase-like protein	W7T9K1_9STRA	67 kDa	2	-0.11	0.16
P/Control	Triglyceride lipase	W7TSJ6_9STRA	86 kDa	4	-0.18	0.4

Supplementary Table 9. Differential expression of proteins annotated as polyketide synthase in N-/Control and P-/Control treatments. None were determined significantly differently expressed either at the Benjamini-Hochberg adjusted p-value thresholds, or at the 5% level.

Condition	Name	UniProt Accession Number	Molecular Weight	No. quantified spectra	L_2fc	p-value
N/Control	Cluster of Polyketide synthase	W7U961_9STRA	331 kDa	95	-0.04	0.64
N/Control	Polyketide synthase (Fragment)	I2CR04_NANGC	60 kDa	5	-0.02	0.75
N/Control	Polyketide synthase	W7U9U8_9STRA	171 kDa	3	-0.06	0.42
N/Control	Polyketide synthase	W7U042_9STRA	315 kDa	7	-0.05	0.64
N/Control	Polyketide synthase	W7T9I3_9STRA	69 kDa	5	-0.02	0.75
N/Control	Type i polyketide synthase	W7U1Y5_9STRA	330 kDa	42	0.04	0.065
P/Control	Cluster of Polyketide synthase	W7U961_9STRA	331 kDa	95	0.04	0.17
P/Control	Polyketide synthase (Fragment)	I2CR04_NANGC	60 kDa	5	-0.12	0.24
P/Control	Polyketide synthase	W7U9U8_9STRA	171 kDa	3	-0.09	0.44
P/Control	Polyketide synthase	W7U042_9STRA	315 kDa	7	0.04	0.45
P/Control	Polyketide synthase	W7T9I3_9STRA	69 kDa	5	0.02	0.75
P/Control	Type i polyketide synthase	W7U1Y5_9STRA	330 kDa	42	0.03	0.84
